# Subject Index Volume 8

**DOI:** 10.1111/jdi.12744

**Published:** 2017-11-03

adipocyte fatty acid‐binding protein

Contribution of serum adipocyte fatty acid‐binding protein levels to the presence of microalbuminuria in a Chinese hyperglycemic population, Hu 582–589

adult‐onset autoimmune diabetes

Chronic hepatitis B virus infection status is more prevalent in patients with type 2 diabetes, Lu 619–625

advanced glycation end‐products

Advanced glycation end‐products are a risk for muscle weakness in Japanese patients with type 1 diabetes, Mori 377–382

albuminuria

Sarcopenia is associated with incident albuminuria in patients with type 2 diabetes: A retrospective observational study, Bouchi 783–787

amniotic fluid‐derived stem cells

Enhanced differentiation of human amniotic fluid‐derived stem cells into insulin‐producing cells *in vitro,* Mu 34–43

anagliptin

Mechanism of lipid‐lowering action of the dipeptidyl peptidase‐4 inhibitor, anagliptin, in low‐density lipoprotein receptor‐deficient mice, Wataru Yano 155–160

angiotensin‐(1–7)

Angiotensin‐(1–7) protects cardiomyocytes against high glucose‐induced injuries through inhibiting reactive oxygen species‐activated leptin–p38 mitogen‐activated protein kinase/extracellular signal‐regulated protein kinase 1/2 pathways, but not the leptin–c‐Jun N‐terminal kinase pathway *in* vitro, Lei 434–445

anti‐glutamic acid decarboxylase antibody

Diagnostic accuracy of the anti‐glutamic acid decarboxylase antibody in type 1 diabetes mellitus: Comparison between radioimmunoassay and enzyme‐linked immunosorbent assay, Murata 475–479

Asia

Efficacy and safety of premixed insulin analogs in Asian patients with type 2 diabetes: A systematic review, Sheu 518–534

average age at the time of death

Causes of death in Japanese patients with diabetes based on the results of a survey of 45,708 cases during 2001–2010: Report of the Committee on Causes of Death in Diabetes Mellitus, Nakamura 397–410

β‐cell function

Analysis of the association between glucose profiles and β‐cell function for diabetic cardiovascular autonomic neuropathy in China, Fang 354–362

β‐cell proliferation

Mild hyperglycemia triggered islet function recovery in streptozotocin‐induced insulin‐deficient diabetic rats, Cheng 44–55

basal–bolus insulin therapy

Preventive effect of ipragliflozin on nocturnal hypoglycemia in patients with type 2 diabetes treated with basal‐bolus insulin therapy: An open‐label, single‐center, parallel, randomized control study, Okajima 341–345

body mass index

Reported prevalence of gestational diabetes in Scotland: The relationship with obesity, age, socioeconomic status, smoking and macrosomia, and how many are we missing?, Collier 161–167

bodyweight

Comparisons of weight changes between sodium‐glucose cotransporter 2 inhibitors treatment and glucagon‐like peptide‐1 analogs treatment in type 2 diabetes patients: A meta‐analysis, Cai 510–517

bolus calculator

Effect of carbohydrate counting using bolus calculators on glycemic control in type 1 diabetes patients during continuous subcutaneous insulin infusion, Yamada 496–500

brachial‐ankle pulse wave velocity

Brachial‐ankle pulse wave velocity, but not ankle‐brachial index, predicts all‐cause mortality in patients with diabetes after lower extremity amputation, Kazuki Ikura 250–253

C‐reactive protein

Association of type 2 diabetes and an inflammatory marker with incident bone fracture among a Japanese cohort, Mitama 709–715

cardiomyocytes

Angiotensin‐(1–7) protects cardiomyocytes against high glucose‐induced injuries through inhibiting reactive oxygen species‐activated leptin–p38 mitogen‐activated protein kinase/extracellular signal‐regulated protein kinase 1/2 pathways, but not the leptin–c‐Jun N‐terminal kinase pathway *in* vitro, Lei 434–445

cardiovascular disease

Prescription of oral hypoglycemic agents for patients with type 2 diabetes mellitus: A retrospective cohort study using a Japanese hospital database, Tanabe 227–234
Impact of postprandial hyperglycemia at clinic visits on the incidence of cardiovascular events and all‐cause mortality in patients with type 2 diabetes, Takao 600–608

cardiovascular risk

Vildagliptin reduces plasma stromal cell‐derived factor‐1α in patients with type 2 diabetes compared with glimepiride, Park 218–226

carotid artery intima‐media thickness

High neutrophil‐to‐lymphocyte ratio is associated with increased carotid artery intima‐media thickness in type 2 diabetes, Li 101–107
Association between carotid intima‐media thickness and fasting blood glucose level: A population‐based cross‐sectional study among low‐income adults in rural China, Gao 788–797

causes of death in Japanese diabetics

Causes of death in Japanese patients with diabetes based on the results of a survey of 45,708 cases during 2001–2010: Report of the Committee on Causes of Death in Diabetes Mellitus, Nakamura 397–410

cerebral large and small vessel disease

Pathogenesis and neuroimaging of cerebral large and small vessel disease in type 2 diabetes: A possible link between cerebral and retinal microvascular abnormalities, Umemura 134–148

children

Population‐based cross‐sectional study on insulin resistance and insulin‐secretory capacity in Japanese school children, Nishimura 672–676

clinical

Pioglitazone improves whole‐body aerobic capacity and skeletal muscle energy metabolism in patients with metabolic syndrome, Yokota 535–541
Review of insulin‐associated hypoglycemia and its impact on the management of diabetes in Southeast Asian countries, Goh 635–645

Commentary

Novel mechanism for counter‐regulatory responses to hypoglycemia, Himeno 29–31
Salt and sugar: Bad company, Wada 32–33
Glucose‐ or insulin resistance‐mediated β‐cell replication: PKCζ integrates the proliferative signalling, Shirakawa 149–151
Where does liver fat go? A possible molecular link between fatty liver and diabetes, Akihiro Kikuchi 152–154
Cardiovascular safety trials of incretin‐based drugs: What do they mean?, Yabe 272–276
Retinopathy: A sign of cerebral small vessel disease in diabetes?, Toshitaka Umemura 428–430
Lessons from a cardiovascular outcome trial with liraglutide in type 2 diabetes, Kim 431–433
Heterogeneity of β‐cells, Kume 656–657
Impact of empagliflozin on diabetic kidney disease, Koya 658–660
Increased trend in the incidence of diabetes among youths in the USA during 2002–2012, Urakami 748–749

continuous glucose monitoring

Determinants of hemoglobin A1c level in patients with type 2 diabetes after in‐hospital diabetes education: A study based on continuous glucose monitoring, Torimoto 314–320
Relationship of glycated hemoglobin, and fasting and postprandial hyperglycemia in type 2 diabetes mellitus patients in Malaysia, Lim 453–461

continuous subcutaneous insulin infusion

Effect of carbohydrate counting using bolus calculators on glycemic control in type 1 diabetes patients during continuous subcutaneous insulin infusion, Yamada 496–500

cyclic adenosine monophosphate response element binding protein

Roles of p300 and cyclic adenosine monophosphate response element binding protein in high glucose‐induced hypoxia‐inducible factor 1α inactivation under hypoxic conditions, Ding 277–285

cytokines

Adiponectin and pro‐inflammatory cytokines are modulated in Vietnamese patients with type 2 diabetes mellitus, Tong 295–305

deoxyribonucleic methylation

Associations between changes in the maternal gut microbiome and differentially methylated regions of diabetes‐associated genes in fetuses: A pilot study from a birth cohort study, Tachibana 550–553

diabetes

Ghrelin is a possible new predictor associated with executive function in patients with type 2 diabetes mellitus, Chen 306–313
Prevalence and characteristics of *Staphylococcus aureus*and methicillin‐resistant *Staphylococcus aureus* nasal colonization among a community‐based diabetes population in Foshan, China, Lin 383–391
Heterogeneity of glycometabolism in patients with gestational diabetes mellitus: Retrospective study of 1,683 pregnant women, Yan 554–559
Diabetes, hypertension, obesity, and long‐term risk of renal disease mortality: Racial and socioeconomic differences, Lankarani 590–599
Are home visits an effective method for diabetes management? A quantitative systematic review and meta‐analysis, Han 701–708
Forkhead box class O family member proteins: The biology and pathophysiological roles in diabetes, Tsuchiya 726–734

diabetes mellitus

Enhanced differentiation of human amniotic fluid‐derived stem cells into insulin‐producing cells *in vitro,* Mu 34–43
Factors influencing inter‐day glycemic variability in diabetic outpatients receiving insulin therapy, Mori 69–74
Impact of diet on the efficacy of insulin lispro mix 25 and insulin lispro mix 50 as starter insulin in East Asian patients with type 2 diabetes: Subgroup analysis of the Comparison Between Low Mixed Insulin and Mid Mixed Insulin as Starter Insulin For Patients with Type 2 Diabetes Mellitus (CLASSIFY Study) randomized trial, Chen 75–83
Varenicline in smokers with diabetes: A pooled analysis of 15 randomized, placebo‐controlled studies of varenicline, Tonstad 93–100
New glycemic targets for patients with diabetes from the Japan Diabetes Society, Araki 123–125
Determinants of hemoglobin A1c level in patients with type 2 diabetes after in‐hospital diabetes education: A study based on continuous glucose monitoring, Torimoto 314–320
Reversible splenial lesion syndrome with a hyperosmolar hyperglycemic state and neuroleptic malignant syndrome caused by olanzapine, Kaino 392–394
Previous dropout from diabetic care as a predictor of patients’ willingness to use mobile applications for self‐management: A cross‐sectional study, Yamaguchi 542–549
Association between particulate matter 2.5 and diabetes mellitus: A meta‐analysis of cohort studies, He 687–696
Association of type 2 diabetes and an inflammatory marker with incident bone fracture among a Japanese cohort, Mitama 709–715
Impact of non‐alcoholic fatty pancreas disease on glucose metabolism, Yu 735–747

diabetes therapy

Duration of diabetes and types of diabetes therapy in Japanese patients with type 2 diabetes: The Japan Diabetes Complication and its Prevention prospective study 3 (JDCP study 3), Hayashino 243–249

diabetic burn wound healing

Roles of p300 and cyclic adenosine monophosphate response element binding protein in high glucose‐induced hypoxia‐inducible factor 1α inactivation under hypoxic conditions, Ding 277–285

diabetic cardiovascular autonomic neuropathy

Analysis of the association between glucose profiles and β‐cell function for diabetic cardiovascular autonomic neuropathy in China, Fang 354–362

diabetic kidney disease

Clinical predictive factors in diabetic kidney disease progression, Radcliffe 6–18
Mechanisms of metabolic memory and renal hypoxia as a therapeutic target in diabetic kidney disease, Hirakawa 261–271

diabetic nephropathy

Clinical predictive factors in diabetic kidney disease progression, Radcliffe 6–18
Effects of mineralocorticoid receptor antagonists on the progression of diabetic nephropathy, Sun 609–618

diabetic neuropathy

Association of type 2 diabetes susceptibility loci with peripheral nerve function in a Chinese population with diabetes, Lu 115–120
Four‐year sequential nerve conduction changes since first visit in Japanese patients with early type 2 diabetes, Kobori 369–376

diabetic retinopathy

Pathogenesis and neuroimaging of cerebral large and small vessel disease in type 2 diabetes: A possible link between cerebral and retinal microvascular abnormalities, Umemura 134–148

diet

Impact of diet on the efficacy of insulin lispro mix 25 and insulin lispro mix 50 as starter insulin in East Asian patients with type 2 diabetes: Subgroup analysis of the Comparison Between Low Mixed Insulin and Mid Mixed Insulin as Starter Insulin For Patients with Type 2 Diabetes Mellitus (CLASSIFY Study) randomized trial, Chen 75–83

dipeptidyl peptidase‐4 inhibitors

Single and multiple dose pharmacokinetics and pharmacodynamics of omarigliptin, a novel, once‐weekly dipeptidyl peptidase‐4 inhibitor, in healthy Japanese men, Saori Tsuchiya 84–92
Mechanism of lipid‐lowering action of the dipeptidyl peptidase‐4 inhibitor, anagliptin, in low‐density lipoprotein receptor‐deficient mice, Wataru Yano 155–160

disease susceptibility

Nucleotide substitutions in *CD101*, the human homolog of a diabetes susceptibility gene in non‐obese diabetic mouse, in patients with type 1 diabetes, Okuno 286–294

distal symmetric polyneuropathy

Sudoscan is an effective screening method for asymptomatic diabetic neuropathy in Chinese type 2 diabetes mellitus patients, Mao 363–368

donor liver steatosis

New‐onset diabetes after transplantation
Donor liver steatosis: A risk factor for early new‐onset diabetes after liver transplantation, Xue 181–187

durability

Duration of diabetes and types of diabetes therapy in Japanese patients with type 2 diabetes: The Japan Diabetes Complication and its Prevention prospective study 3 (JDCP study 3), Hayashino 243–249

duration of diabetes

Duration of diabetes and types of diabetes therapy in Japanese patients with type 2 diabetes: The Japan Diabetes Complication and its Prevention prospective study 3 (JDCP study 3), Hayashino 243–249

Editorial

Continuous glucose monitoring in China: Then, now and in the future, Jia 3–5
Managing non‐alcoholic fatty liver disease in diabetes: Challenges and opportunities, Lee 131–133
Integration of diffusion of innovation theory into diabetes care, Lien 259–260
Diabetes and pancreas size, does it matter?, Yagihashi 413–415
Evaluation of large‐scale clinical trials on cardiovascular disease risk in patients with type 2 diabetes mellitus treated with dipeptidyl peptidase 4 inhibitors and a new class of drugs, Kurose 633–634
Impaired insulin clearance as a cause rather than a consequence of insulin resistance, Watada 723–725

elderly

Committee Report: Glycemic targets for elderly patients with diabetes, Japan Diabetes Society (JDS)/Japan Geriatrics Society (JGS) Joint Committee on Improving Care for Elderly Patients with Diabetes 126–128
Safety and effectiveness of tofogliflozin in elderly Japanese patients with type 2 diabetes mellitus: A post‐marketing study (J‐STEP/EL Study), Utsunomiya 766–775

endothelial function

Linagliptin improves endothelial function in patients with type 2 diabetes: A randomized study of linagliptin effectiveness on endothelial function, Shigiyama 330–340

enzyme‐linked immunosorbent assay

Diagnostic accuracy of the anti‐glutamic acid decarboxylase antibody in type 1 diabetes mellitus: Comparison between radioimmunoassay and enzyme‐linked immunosorbent assay, Murata 475–479

executive function

Ghrelin is a possible new predictor associated with executive function in patients with type 2 diabetes mellitus, Chen 306–313

exome

Nucleotide substitutions in *CD101*, the human homolog of a diabetes susceptibility gene in non‐obese diabetic mouse, in patients with type 1 diabetes, Okuno 286–294

fasting blood glucose

Association between carotid intima‐media thickness and fasting blood glucose level: A population‐based cross‐sectional study among low‐income adults in rural China, Gao 788–797

fasting hyperglycemia

Relationship of glycated hemoglobin, and fasting and postprandial hyperglycemia in type 2 diabetes mellitus patients in Malaysia, Lim 453–461

fatty acid oxidation

Low glucose induces mitochondrial reactive oxygen species via fatty acid oxidation in bovine aortic endothelial cells, Kajihara 750–761

ferritin

Circulating ferritin concentrations and risk of type 2 diabetes in Japanese individuals, Akter 462–470

forkhead box class O family member proteins

Forkhead box class O family member proteins: The biology and pathophysiological roles in diabetes, Tsuchiya 726–734

genetics

Association of type 2 diabetes susceptibility loci with peripheral nerve function in a Chinese population with diabetes, Lu 115–120

genetic susceptibility

Rare human leukocyte antigen genotype in two siblings with type 1 diabetes in a Japanese family clustered with type 1 diabetes, Ina 762–765

gestational diabetes mellitus

Reported prevalence of gestational diabetes in Scotland: The relationship with obesity, age, socioeconomic status, smoking and macrosomia, and how many are we missing?, Collier 161–167
Neck circumference might predict gestational diabetes mellitus in Han Chinese women: A nested case–control study, He 168–173
Association between *TCF7L2* polymorphisms and gestational diabetes mellitus: A meta‐analysis, Chang 560–570
Circulating milk fat globule‐epidermal growth factor 8 levels are increased in pregnancy and gestational diabetes mellitus, Li 571–581
Higher maternal serum prolactin levels are associated with reduced glucose tolerance during pregnancy, Ekinci 697–700

ghrelin

Ghrelin is a possible new predictor associated with executive function in patients with type 2 diabetes mellitus, Chen 306–313

glucagon‐like peptide‐1 analogs

Comparisons of weight changes between sodium‐glucose cotransporter 2 inhibitors treatment and glucagon‐like peptide‐1 analogs treatment in type 2 diabetes patients: A meta‐analysis, Cai 510–517

glucose profile

Analysis of the association between glucose profiles and β‐cell function for diabetic cardiovascular autonomic neuropathy in China, Fang 354–362

glucose tolerance

Plasma mannose level, a putative indicator of glycogenolysis, and glucose tolerance in Japanese individuals, Yoshimura 489–495

glycemic targets

Committee Report: Glycemic targets for elderly patients with diabetes, Japan Diabetes Society (JDS)/Japan Geriatrics Society (JGS) Joint Committee on Improving Care for Elderly Patients with Diabetes 126–128

glycogenolysis

Plasma mannose level, a putative indicator of glycogenolysis, and glucose tolerance in Japanese individuals, Yoshimura 489–495

HbA1c

New glycemic targets for patients with diabetes from the Japan Diabetes Society, Araki 123–125

hepatitis B virus

Chronic hepatitis B virus infection status is more prevalent in patients with type 2 diabetes, Lu 619–625

heterogeneity

Heterogeneity of glycometabolism in patients with gestational diabetes mellitus: Retrospective study of 1,683 pregnant women, Yan 554–559

high glucose

Angiotensin‐(1–7) protects cardiomyocytes against high glucose‐induced injuries through inhibiting reactive oxygen species‐activated leptin–p38 mitogen‐activated protein kinase/extracellular signal‐regulated protein kinase 1/2 pathways, but not the leptin–c‐Jun N‐terminal kinase pathway *in* vitro, Lei 434–445

home visit

Are home visits an effective method for diabetes management? A quantitative systematic review and meta‐analysis, Han 701–708

human leukocyte antigen haplotype

Rare human leukocyte antigen genotype in two siblings with type 1 diabetes in a Japanese family clustered with type 1 diabetes, Ina 762–765

hypoglycemia

Review of insulin‐associated hypoglycemia and its impact on the management of diabetes in Southeast Asian countries, Goh 635–645
Diabetes management and daily functioning burden of non‐severe hypoglycemia in Japanese people treated with insulin, Ohashi 776–782

incident bone fracture

Association of type 2 diabetes and an inflammatory marker with incident bone fracture among a Japanese cohort, Mitama 709–715

inflammation

Circulating milk fat globule‐epidermal growth factor 8 levels are increased in pregnancy and gestational diabetes mellitus, Li 571–581

in‐hospital diabetes education

Determinants of hemoglobin A1c level in patients with type 2 diabetes after in‐hospital diabetes education: A study based on continuous glucose monitoring, Torimoto 314–320

insulin

Sitagliptin added to stable insulin therapy with or without metformin in Chinese patients with type 2 diabetes, Shankar 321–329
Review of insulin‐associated hypoglycemia and its impact on the management of diabetes in Southeast Asian countries, Goh 635–645

insulin deficiency

Population‐based cross‐sectional study on insulin resistance and insulin‐secretory capacity in Japanese school children, Nishimura 672–676

insulin degludec/insulin aspart

Insulin degludec/insulin aspart vs biphasic insulin aspart 30 twice daily in Japanese patients with type 2 diabetes: A randomized controlled trial, Yukiko Onishi 210–217

insulin‐like growth factor‐1

Elevated expression levels of serum insulin‐like growth factor‐1, tumor necrosis factor‐α and vascular endothelial growth factor 165 might exacerbate type 2 diabetic nephropathy, Xiang Li 108–114

insulin lispro

Impact of diet on the efficacy of insulin lispro mix 25 and insulin lispro mix 50 as starter insulin in East Asian patients with type 2 diabetes: Subgroup analysis of the Comparison Between Low Mixed Insulin and Mid Mixed Insulin as Starter Insulin For Patients with Type 2 Diabetes Mellitus (CLASSIFY Study) randomized trial, Chen 75–83

insulin‐producing cells

Enhanced differentiation of human amniotic fluid‐derived stem cells into insulin‐producing cells *in vitro,* Mu 34–43

insulin resistance

Population‐based cross‐sectional study on insulin resistance and insulin‐secretory capacity in Japanese school children, Nishimura 672–676

insulin secretion

Undercarboxylated osteocalcin can predict insulin secretion ability in type 2 diabetes, Takashi 471–474

insulin signaling

Forkhead box class O family member proteins: The biology and pathophysiological roles in diabetes, Tsuchiya 726–734

insulin therapy

Delay of insulin initiation in patients with type 2 diabetes mellitus inadequately controlled with oral hypoglycemic agents (analysis of patient‐ and physician‐related factors): A prospective observational DIPP‐FACTOR study in Korea, Kim 346–353

interaction

Analysis of risk factors and their interactions in type 2 diabetes mellitus: A cross‐sectional survey in Guilin, China, Disha Zou 188–194

islet function recovery

Mild hyperglycemia triggered islet function recovery in streptozotocin‐induced insulin‐deficient diabetic rats, Cheng 44–55

Japanese

Insulin degludec/insulin aspart vs biphasic insulin aspart 30 twice daily in Japanese patients with type 2 diabetes: A randomized controlled trial, Yukiko Onishi 210–217
Circulating ferritin concentrations and risk of type 2 diabetes in Japanese individuals, Akter 462–470

Kumamoto Declaration 2013

New glycemic targets for patients with diabetes from the Japan Diabetes Society, Araki 123–125

Letter to the Editor

Novel subtype of congenital partial lipodystrophy with mandibular hypoplasia, sensorineural deafness and short stature of unknown genetic origin, Sasaki 121–122
Changes in carotid intima‐media thickening in patients with type 2 diabetes mellitus: Subanalysis of the Sitagliptin Preventive Study of Intima‐Media Thickness Evaluation, Mita 254–255
Advanced breast cancer in a relatively young man with severe obesity and type 2 diabetes mellitus, Obata 395–396
Acute‐onset type 1 diabetes mellitus caused by nivolumab in a patient with advanced pulmonary adenocarcinoma, Kumagai 798–799

lifestyle

Factors influencing inter‐day glycemic variability in diabetic outpatients receiving insulin therapy, Mori 69–74

linagliptin

Pharmacokinetic and pharmacodynamic evaluation of linagliptin for the treatment of type 2 diabetes mellitus, with consideration of Asian patient populations, Ceriello 19–28
Linagliptin improves endothelial function in patients with type 2 diabetes: A randomized study of linagliptin effectiveness on endothelial function, Shigiyama 330–340

lipid metabolism

Mechanism of lipid‐lowering action of the dipeptidyl peptidase‐4 inhibitor, anagliptin, in low‐density lipoprotein receptor‐deficient mice, Wataru Yano 155–160
Selective peroxisome proliferator‐activated receptor‐α modulator K‐877 efficiently activates the peroxisome proliferator‐activated receptor‐α pathway and improves lipid metabolism in mice, Takei 446–452

liver transplantation

Donor liver steatosis: A risk factor for early new‐onset diabetes after liver transplantation, Xue 181–187

Long‐Evans Agouti rat

Proteomic analysis of serum biomarkers for prediabetes using the Long‐Evans Agouti rat, a spontaneous animal model of type 2 diabetes mellitus, Takahashi 661–671

lower extremity amputation

Brachial‐ankle pulse wave velocity, but not ankle‐brachial index, predicts all‐cause mortality in patients with diabetes after lower extremity amputation, Kazuki Ikura 250–253

low glucose

Low glucose induces mitochondrial reactive oxygen species via fatty acid oxidation in bovine aortic endothelial cells, Kajihara 750–761

macrosomia

Reported prevalence of gestational diabetes in Scotland: The relationship with obesity, age, socioeconomic status, smoking and macrosomia, and how many are we missing?, Collier 161–167

malignant neoplasia

Causes of death in Japanese patients with diabetes based on the results of a survey of 45,708 cases during 2001–2010: Report of the Committee on Causes of Death in Diabetes Mellitus, Nakamura 397–410

mannose

Plasma mannose level, a putative indicator of glycogenolysis, and glucose tolerance in Japanese individuals, Yoshimura 489–495

maternal glycemia

Higher maternal serum prolactin levels are associated with reduced glucose tolerance during pregnancy, Ekinci 697–700

mean of daily difference

Factors influencing inter‐day glycemic variability in diabetic outpatients receiving insulin therapy, Mori 69–74

meta‐analysis

Specific types of alcoholic beverage consumption and risk of type 2 diabetes: A systematic review and meta‐analysis, Huang 56–68
Effects of yoga in adults with type 2 diabetes mellitus: A meta‐analysis, Cui 201–209
Association between omega‐3 fatty acids consumption and the risk of type 2 diabetes: A meta‐analysis of cohort studies, Chen 480–488
Unstable bodyweight and incident type 2 diabetes mellitus: A meta‐analysis, Kodama 501–509
Effects of mineralocorticoid receptor antagonists on the progression of diabetic nephropathy, Sun 609–618
Association between particulate matter 2.5 and diabetes mellitus: A meta‐analysis of cohort studies, He 687–696
Are home visits an effective method for diabetes management? A quantitative systematic review and meta‐analysis, Han 701–708

metabolic memory

Mechanisms of metabolic memory and renal hypoxia as a therapeutic target in diabetic kidney disease, Hirakawa 261–271

metabolic syndrome

Neck circumference might predict gestational diabetes mellitus in Han Chinese women: A nested case–control study, He 168–173
Pioglitazone improves whole‐body aerobic capacity and skeletal muscle energy metabolism in patients with metabolic syndrome, Yokota 535–541
Peripheral neuropathy in prediabetes and the metabolic syndrome, Stino 646–655
Impact of non‐alcoholic fatty pancreas disease on glucose metabolism, Yu 735–747

methicillin‐resistant *Staphylococcus aureus*

Prevalence and characteristics of *Staphylococcus aureus*and methicillin‐resistant *Staphylococcus aureus* nasal colonization among a community‐based diabetes population in Foshan, China, Lin 383–391

mHealth

Previous dropout from diabetic care as a predictor of patients’ willingness to use mobile applications for self‐management: A cross‐sectional study, Yamaguchi 542–549

microalbuminuria

Contribution of serum adipocyte fatty acid‐binding protein levels to the presence of microalbuminuria in a Chinese hyperglycemic population, Hu 582–589

microangiopathy

The number of microvascular complications is associated with an increased risk for severity of periodontitis in type 2 diabetes patients: Results of a multicenter hospital‐based cross‐sectional study, Nitta 677–686

microbiome

Associations between changes in the maternal gut microbiome and differentially methylated regions of diabetes‐associated genes in fetuses: A pilot study from a birth cohort study, Tachibana 550–553

mild hyperglycemia

Mild hyperglycemia triggered islet function recovery in streptozotocin‐induced insulin‐deficient diabetic rats, Cheng 44–55

milk‐fat globule‐epidermal growth factor 8

Circulating milk fat globule‐epidermal growth factor 8 levels are increased in pregnancy and gestational diabetes mellitus, Li 571–581

mineralocorticoid receptor antagonist

Effects of mineralocorticoid receptor antagonists on the progression of diabetic nephropathy, Sun 609–618

mitochondrial reactive oxygen species

Low glucose induces mitochondrial reactive oxygen species via fatty acid oxidation in bovine aortic endothelial cells, Kajihara 750–761

mixed connective tissue disease

Successful treatment of type B insulin resistance with mixed connective tissue disease by pulse glucocorticoids and cyclophosphamide, Yang 626–628

mortality

Brachial‐ankle pulse wave velocity, but not ankle‐brachial index, predicts all‐cause mortality in patients with diabetes after lower extremity amputation, Kazuki Ikura 250–253
Impact of postprandial hyperglycemia at clinic visits on the incidence of cardiovascular events and all‐cause mortality in patients with type 2 diabetes, Takao 600–608

mutation

Nucleotide substitutions in *CD101*, the human homolog of a diabetes susceptibility gene in non‐obese diabetic mouse, in patients with type 1 diabetes, Okuno 286–294

natural history

Four‐year sequential nerve conduction changes since first visit in Japanese patients with early type 2 diabetes, Kobori 369–376

neck circumference

Neck circumference might predict gestational diabetes mellitus in Han Chinese women: A nested case–control study, He 168–173

neonatal diabetes

First case of neonatal diabetes with *KCNJ11* Q52R mutation successfully switched from insulin to sulphonylurea treatment, Ioacara 716–719

nerve conduction

Four‐year sequential nerve conduction changes since first visit in Japanese patients with early type 2 diabetes, Kobori 369–376

nerve conduction study

Association of type 2 diabetes susceptibility loci with peripheral nerve function in a Chinese population with diabetes, Lu 115–120

neuroleptic malignant syndrome

Reversible splenial lesion syndrome with a hyperosmolar hyperglycemic state and neuroleptic malignant syndrome caused by olanzapine, Kaino 392–394

neutrophil‐to‐lymphocyte ratio

High neutrophil‐to‐lymphocyte ratio is associated with increased carotid artery intima‐media thickness in type 2 diabetes, Li 101–107

new‐onset diabetes after transplantation

Donor liver steatosis: A risk factor for early new‐onset diabetes after liver transplantation, Xue 181–187

night‐time

Diabetes management and daily functioning burden of non‐severe hypoglycemia in Japanese people treated with insulin, Ohashi 776–782

nocturnal hypoglycemia

Preventive effect of ipragliflozin on nocturnal hypoglycemia in patients with type 2 diabetes treated with basal‐bolus insulin therapy: An open‐label, single‐center, parallel, randomized control study, Okajima 341–345

non‐alcoholic fatty liver disease

Deoxyribonucleic acid telomere length shortening can predict the incidence of non‐alcoholic fatty liver disease in patients with type 2 diabetes mellitus, Ping 174–180

non‐alcoholic fatty pancreas disease

Impact of non‐alcoholic fatty pancreas disease on glucose metabolism, Yu 735–747

non‐severe

Diabetes management and daily functioning burden of non‐severe hypoglycemia in Japanese people treated with insulin, Ohashi 776–782

nutritional guidance

Effect of a newly‐devised nutritional guide based on self‐efficacy for patients with type 2 diabetes in Japan over 2 years: 1‐year intervention and 1‐year follow‐up studies, Yamamoto 195–200

olanzapine

Reversible splenial lesion syndrome with a hyperosmolar hyperglycemic state and neuroleptic malignant syndrome caused by olanzapine, Kaino 392–394

omarigliptin

Single and multiple dose pharmacokinetics and pharmacodynamics of omarigliptin, a novel, once‐weekly dipeptidyl peptidase‐4 inhibitor, in healthy Japanese men, Saori Tsuchiya 84–92

omega‐3 fatty acids

Association between omega‐3 fatty acids consumption and the risk of type 2 diabetes: A meta‐analysis of cohort studies, Chen 480–488

once‐weekly

Single and multiple dose pharmacokinetics and pharmacodynamics of omarigliptin, a novel, once‐weekly dipeptidyl peptidase‐4 inhibitor, in healthy Japanese men, Saori Tsuchiya 84–92

oral hypoglycemic agent

Prescription of oral hypoglycemic agents for patients with type 2 diabetes mellitus: A retrospective cohort study using a Japanese hospital database, Tanabe 227–234

oral hypoglycemic drugs

Metabolic and hemodynamic effects of sodium‐dependent glucose cotransporter 2 inhibitors on cardio‐renal protection in the treatment of patients with type 2 diabetes mellitus, Kashiwagi 416–427

overweight

Adiponectin and pro‐inflammatory cytokines are modulated in Vietnamese patients with type 2 diabetes mellitus, Tong 295–305

P300

Roles of p300 and cyclic adenosine monophosphate response element binding protein in high glucose‐induced hypoxia‐inducible factor 1α inactivation under hypoxic conditions, Ding 277–285

particulate matter 2.5

Association between particulate matter 2.5 and diabetes mellitus: A meta‐analysis of cohort studies, He 687–696

pediatric diabetes

First case of neonatal diabetes with *KCNJ11* Q52R mutation successfully switched from insulin to sulphonylurea treatment, Ioacara 716–719

periodontitis

The number of microvascular complications is associated with an increased risk for severity of periodontitis in type 2 diabetes patients: Results of a multicenter hospital‐based cross‐sectional study, Nitta 677–686

peripheral neuropathy

Peripheral neuropathy in prediabetes and the metabolic syndrome, Stino 646–655

peroxisome proliferator‐activated receptor‐α

Selective peroxisome proliferator‐activated receptor‐α modulator K‐877 efficiently activates the peroxisome proliferator‐activated receptor‐α pathway and improves lipid metabolism in mice, Takei 446–452

pharmacodynamics

Pharmacokinetic and pharmacodynamic evaluation of linagliptin for the treatment of type 2 diabetes mellitus, with consideration of Asian patient populations, Ceriello 19–28

pharmacokinetics

Pharmacokinetic and pharmacodynamic evaluation of linagliptin for the treatment of type 2 diabetes mellitus, with consideration of Asian patient populations, Ceriello 19–28

physician and patient behaviors

Delay of insulin initiation in patients with type 2 diabetes mellitus inadequately controlled with oral hypoglycemic agents (analysis of patient‐ and physician‐related factors): A prospective observational DIPP‐FACTOR study in Korea, Kim 346–353

polymorphism

Association between *TCF7L2* polymorphisms and gestational diabetes mellitus: A meta‐analysis, Chang 560–570

postprandial blood glucose

Impact of postprandial hyperglycemia at clinic visits on the incidence of cardiovascular events and all‐cause mortality in patients with type 2 diabetes, Takao 600–608

postprandial hyperglycemia

Relationship of glycated hemoglobin, and fasting and postprandial hyperglycemia in type 2 diabetes mellitus patients in Malaysia, Lim 453–461

prediabetes

Peripheral neuropathy in prediabetes and the metabolic syndrome, Stino 646–655

pregnancy

Heterogeneity of glycometabolism in patients with gestational diabetes mellitus: Retrospective study of 1,683 pregnant women, Yan 554–559
Higher maternal serum prolactin levels are associated with reduced glucose tolerance during pregnancy, Ekinci 697–700

premixed insulin

Efficacy and safety of premixed insulin analogs in Asian patients with type 2 diabetes: A systematic review, Sheu 518–534

quality of life

Psychometric properties of the Chinese version of the Problem Areas in Diabetes scale (SG‐PAID‐C) among high‐risk polypharmacy patients with uncontrolled type 2 diabetes in Singapore, Siaw 235–242

quantitative serum proteomics

Proteomic analysis of serum biomarkers for prediabetes using the Long‐Evans Agouti rat, a spontaneous animal model of type 2 diabetes mellitus, Takahashi 661–671

renal diseases

Diabetes, hypertension, obesity, and long‐term risk of renal disease mortality: Racial and socioeconomic differences, Lankarani 590–599

renal hypoxia

Mechanisms of metabolic memory and renal hypoxia as a therapeutic target in diabetic kidney disease, Hirakawa 261–271

risk factor

Analysis of risk factors and their interactions in type 2 diabetes mellitus: A cross‐sectional survey in Guilin, China, Disha Zou 188–194

risk factors

Clinical predictive factors in diabetic kidney disease progression, Radcliffe 6–18
Association between carotid intima‐media thickness and fasting blood glucose level: A population‐based cross‐sectional study among low‐income adults in rural China, Gao 788–797

sarcopenia

Advanced glycation end‐products are a risk for muscle weakness in Japanese patients with type 1 diabetes, Mori 377–382
Sarcopenia is associated with incident albuminuria in patients with type 2 diabetes: A retrospective observational study, Bouchi 783–787

screening

Sudoscan is an effective screening method for asymptomatic diabetic neuropathy in Chinese type 2 diabetes mellitus patients, Mao 363–368

selective peroxisome proliferator‐activated receptor‐α modulator

Selective peroxisome proliferator‐activated receptor‐α modulator K‐877 efficiently activates the peroxisome proliferator‐activated receptor‐α pathway and improves lipid metabolism in mice, Takei 446–452

self‐management

Effect of a newly‐devised nutritional guide based on self‐efficacy for patients with type 2 diabetes in Japan over 2 years: 1‐year intervention and 1‐year follow‐up studies, Yamamoto 195–200
Previous dropout from diabetic care as a predictor of patients’ willingness to use mobile applications for self‐management: A cross‐sectional study, Yamaguchi 542–549

serine protease inhibitor A3

Proteomic analysis of serum biomarkers for prediabetes using the Long‐Evans Agouti rat, a spontaneous animal model of type 2 diabetes mellitus, Takahashi 661–671

sitagliptin

Sitagliptin added to stable insulin therapy with or without metformin in Chinese patients with type 2 diabetes, Shankar 321–329

smoking

Varenicline in smokers with diabetes: A pooled analysis of 15 randomized, placebocontrolled studies of varenicline, Tonstad 93–100

social class

Diabetes, hypertension, obesity, and long‐term risk of renal disease mortality: Racial and socioeconomic differences, Lankarani 590–599

sodium‐glucose transporter 2

Safety and effectiveness of tofogliflozin in elderly Japanese patients with type 2 diabetes mellitus: A post‐marketing study (J‐STEP/EL Study), Utsunomiya 766–775

sodium‐glucose co‐transporter 2 inhibitor

Preventive effect of ipragliflozin on nocturnal hypoglycemia in patients with type 2 diabetes treated with basal‐bolus insulin therapy: An open‐label, single‐center, parallel, randomized control study, Okajima 341–345

sodium‐glucose cotransporter 2 inhibitors

Metabolic and hemodynamic effects of sodium‐dependent glucose cotransporter 2 inhibitors on cardio‐renal protection in the treatment of patients with type 2 diabetes mellitus, Kashiwagi 416–427
Comparisons of weight changes between sodium‐glucose cotransporter 2 inhibitors treatment and glucagon‐like peptide‐1 analogs treatment in type 2 diabetes patients: A meta‐analysis, Cai 510–517

specific alcohol consumption

Specific types of alcoholic beverage consumption and risk of type 2 diabetes: A systematic review and meta‐analysis, Huang 56–68

*Staphylococcus aureus*

Prevalence and characteristics of *Staphylococcus aureus*and methicillin‐resistant *Staphylococcus aureus* nasal colonization among a community‐based diabetes population in Foshan, China, Lin 383–391

stromal cell‐derived factor‐1α

Vildagliptin reduces plasma stromal cell‐derived factor‐1α in patients with type 2 diabetes compared with glimepiride, Park 218–226

sudoscan

Sudoscan is an effective screening method for asymptomatic diabetic neuropathy in Chinese type 2 diabetes mellitus patients, Mao 363–368

sulphonylurea

First case of neonatal diabetes with *KCNJ11* Q52R mutation successfully switched from insulin to sulphonylurea treatment, Ioacara 716–719

*TCF7L2*

Association between *TCF7L2* polymorphisms and gestational diabetes mellitus: A meta‐analysis, Chang 560–570

telomere shortening

Deoxyribonucleic acid telomere length shortening can predict the incidence of non‐alcoholic fatty liver disease in patients with type 2 diabetes mellitus, Ping 174–180

treatment

Successful treatment of type B insulin resistance with mixed connective tissue disease by pulse glucocorticoids and cyclophosphamide, Yang 626–628

treatment drug

Pioglitazone improves whole‐body aerobic capacity and skeletal muscle energy metabolism in patients with metabolic syndrome, Yokota 535–541

type 1 diabetes

Advanced glycation end‐products are a risk for muscle weakness in Japanese patients with type 1 diabetes, Mori 377–382
Effect of carbohydrate counting using bolus calculators on glycemic control in type 1 diabetes patients during continuous subcutaneous insulin infusion, Yamada 496–500
Rare human leukocyte antigen genotype in two siblings with type 1 diabetes in a Japanese family clustered with type 1 diabetes, Ina 762–765

type 1 diabetes mellitus

Diagnostic accuracy of the anti‐glutamic acid decarboxylase antibody in type 1 diabetes mellitus: Comparison between radioimmunoassay and enzyme‐linked immunosorbent assay, Murata 475–479

type 2 diabetes

Specific types of alcoholic beverage consumption and risk of type 2 diabetes: A systematic review and meta‐analysis, Huang 56–68
High neutrophil‐to‐lymphocyte ratio is associated with increased carotid artery intima‐media thickness in type 2 diabetes, Li 101–107
Pathogenesis and neuroimaging of cerebral large and small vessel disease in type 2 diabetes: A possible link between cerebral and retinal microvascular abnormalities, Umemura 134–148
Analysis of risk factors and their interactions in type 2 diabetes mellitus: A cross‐sectional survey in Guilin, China, Disha Zou 188–194
Effect of a newly‐devised nutritional guide based on self‐efficacy for patients with type 2 diabetes in Japan over 2 years: 1‐year intervention and 1‐year follow‐up studies, Yamamoto 195–200
Effects of yoga in adults with type 2 diabetes mellitus: A meta‐analysis, Cui 201–209
Insulin degludec/insulin aspart vs biphasic insulin aspart 30 twice daily in Japanese patients with type 2 diabetes: A randomized controlled trial, Yukiko Onishi 210–217
Linagliptin improves endothelial function in patients with type 2 diabetes: A randomized study of linagliptin effectiveness on endothelial function, Shigiyama 330–340
Delay of insulin initiation in patients with type 2 diabetes mellitus inadequately controlled with oral hypoglycemic agents (analysis of patient‐ and physician‐related factors): A prospective observational DIPP‐FACTOR study in Korea, Kim 346–353
Circulating ferritin concentrations and risk of type 2 diabetes in Japanese individuals, Akter 462–470
Undercarboxylated osteocalcin can predict insulin secretion ability in type 2 diabetes, Takashi 471–474
Association between omega‐3 fatty acids consumption and the risk of type 2 diabetes: A meta‐analysis of cohort studies, Chen 480–488
Chronic hepatitis B virus infection status is more prevalent in patients with type 2 diabetes, Lu 619–625
The number of microvascular complications is associated with an increased risk for severity of periodontitis in type 2 diabetes patients: Results of a multicenter hospital‐based cross‐sectional study, Nitta 677–686

type 2 diabetes mellitus

Deoxyribonucleic acid telomere length shortening can predict the incidence of non‐alcoholic fatty liver disease in patients with type 2 diabetes mellitus, Ping 174–180
Prescription of oral hypoglycemic agents for patients with type 2 diabetes mellitus: A retrospective cohort study using a Japanese hospital database, Tanabe 227–234
Psychometric properties of the Chinese version of the Problem Areas in Diabetes scale (SG‐PAID‐C) among high‐risk polypharmacy patients with uncontrolled type 2 diabetes in Singapore, Siaw 235–242
Adiponectin and pro‐inflammatory cytokines are modulated in Vietnamese patients with type 2 diabetes mellitus, Tong 295–305
Sitagliptin added to stable insulin therapy with or without metformin in Chinese patients with type 2 diabetes, Shankar 321–329
Metabolic and hemodynamic effects of sodium‐dependent glucose cotransporter 2 inhibitors on cardio‐renal protection in the treatment of patients with type 2 diabetes mellitus, Kashiwagi 416–427
Unstable bodyweight and incident type 2 diabetes mellitus: A meta‐analysis, Kodama 501–509
Efficacy and safety of premixed insulin analogs in Asian patients with type 2 diabetes: A systematic review, Sheu 518–534
Sarcopenia is associated with incident albuminuria in patients with type 2 diabetes:A retrospective observational study, Bouchi 783–787
Safety and effectiveness of tofogliflozin in elderly Japanese patients with type 2 diabetes mellitus: A post‐marketing study (J‐STEP/EL Study), Utsunomiya 766–775

type 2 diabetic nephropathy

Elevated expression levels of serum insulin‐like growth factor‐1, tumor necrosis factor‐α and vascular endothelial growth factor 165 might exacerbate type 2 diabetic nephropathy, Xiang Li 108–114

type B insulin resistance

Successful treatment of type B insulin resistance with mixed connective tissue disease by pulse glucocorticoids and cyclophosphamide, Yang 626–628

umbilical cord

Associations between changes in the maternal gut microbiome and differentially methylated regions of diabetes‐associated genes in fetuses: A pilot study from a birth cohort study, Tachibana 550–553

undercarboxylated osteocalcin

Undercarboxylated osteocalcin can predict insulin secretion ability in type 2 diabetes, Takashi 471–474

urinary albumin‐to‐creatinine ratio

Contribution of serum adipocyte fatty acid‐binding protein levels to the presence of microalbuminuria in a Chinese hyperglycemic population, Hu 582–589

validation studies

Psychometric properties of the Chinese version of the Problem Areas in Diabetes scale (SG‐PAID‐C) among high‐risk polypharmacy patients with uncontrolled type 2 diabetes in Singapore, Siaw 235–242

varenicline

Varenicline in smokers with diabetes: A pooled analysis of 15 randomized, placebo‐controlled studies of varenicline, Tonstad 93–100

vascular endothelial growth factor 165

Elevated expression levels of serum insulin‐like growth factor‐1, tumor necrosis factor‐α and vascular endothelial growth factor 165 might exacerbate type 2 diabetic nephropathy, Xiang Li 108–114

vildagliptin

Vildagliptin reduces plasma stromal cell‐derived factor‐1α in patients with type 2 diabetes compared with glimepiride, Park 218–226

weight cycling

Unstable bodyweight and incident type 2 diabetes mellitus: A meta‐analysis, Kodama 501–509

yoga

Effects of yoga in adults with type 2 diabetes mellitus: A meta‐analysis, Cui 201–209

